# A scalable automated framework for multiply-accumulate unit design in high-performance computing applications

**DOI:** 10.1038/s41598-025-26868-3

**Published:** 2025-11-28

**Authors:** Adithya Venkatachalam, Umadevi Seerengasamy, Abraham Sudharson Ponraj

**Affiliations:** 1 School of Electronics Engineering, VIT University, Chennai Campus, Chennai, India; 2 Centre for Nanoelectronics and VLSI Design, School of Electronics Engineering, VIT University, Chennai Campus, Chennai, India

**Keywords:** Multiply-Accumulate, Hardware description Language(HDL), System-on-Chip (SoC), GD multiplier, Carry skip adder, Engineering, Mathematics and computing

## Abstract

Multiply–Accumulate (MAC) units are essential components in real-time Digital Signal Processing (DSP) applications such as image filtering, audio processing, and neural networks, where speed and energy efficiency are critical. This work presents a scalable and automated Hardware Description Language (HDL) generation framework for efficient MAC unit design, enabling faster integration into System-on-Chip (SoC) architectures. A Python automation script is developed to generate synthesizable Verilog code supporting user-defined bit-widths and computation cycles. The framework integrates a Grouping and Decomposition (GD) multiplier with a carry-skip adder, enhancing computational speed and minimizing energy consumption through parallelized data processing. The generated Verilog code is functionally verified using Cadence^®^ Nclaunch, while synthesis and physical implementation are performed using Cadence^®^ Genus and Innovus tools across multiple technology nodes. Experimental evaluation shows that the 8-bit GD-based MAC implemented in 180 nm technology achieves 14.96times lower power, 9.86times improvement in Power Delay Product (PDP) and Energy per Operation (EOP), and 6.52times improvement in Energy Delay Product (EDP) compared to existing designs. Likewise, the 16-bit MAC in 90 nm technology achieves a 70.08% reduction in delay and a 91.2% improvement in EDP. Overall, the proposed framework delivers a scalable, energy-efficient, and automation-driven solution for high-performance MAC design.

## Introduction

Intelligent systems and artificial intelligence (AI) have become pervasive in modern society, with extensive applications across domains such as healthcare, autonomous systems, and the Internet of Things (IoT)^1,2^. However, the increasing demand for AI and machine learning (ML) solutions presents significant issues regarding their performance and scalability^3^. The increasing expectation for prompt and relevant solutions from smart applications is further amplifying this need, thereby requiring the implementation of edge computing^4^. This kind of edge intelligence markedly increases the complexity and performance demands of hardware systems, while functioning within the limitations of conventional embedded environments defined by restricted memory and minimal processing capability. The MAC unit is a crucial computational component of DSP and AI hardware accelerators. MAC units, which execute essential multiply and accumulate operations in algorithms such as convolution, matrix multiplication, and polynomial evaluation, are integral to AI workloads^5^. Conventional fixed-width MAC designs, however, lack the adaptability to accommodate diverse bit-width needs and the optimization necessary for developing AI and ML applications. Current trends in MAC unit design emphasize its integration with machine learning accelerators for artificial intelligence applications. Techniques like data reuse and weight sparsity^6,7^ are being investigated to reduce power consumption and optimize memory bandwidth, hence increasing the significance of MAC units in advanced computing systems.

Over the years, the architecture of MAC units has evolved significantly, maintaining a pivotal role in digital computing, particularly in signal processing and numerical computation domains. The importance of the MAC unit in modern digital systems is highlighted by its progression from simple multiplier and adder components to a unified and efficient integrated MAC architecture. According to recent research, Vedic multiplication methods greatly enhance digital arithmetic processes by lowering hardware resource consumption, increasing speed, and simplifying computations. Scholars emphasize how effective these antiquated techniques are compared to traditional algorithms^8–12^. Parallel processing is made possible by the Urdhva Tiryagbhyam Sutra in particular, which results in circuit designs that are quicker and more effective^8^. These methods are suitable for a range of contemporary digital systems since they are straightforward, power-efficient, modular, and scalable^12^. Studies by^13–16^ on adders concentrate on enhancing the Brent Kung adder for increased speed, less power consumption, and smaller footprint. In order to achieve quicker performance and higher power efficiency than ordinary adders, a hybrid design that combines the Carry Select Adder with Brent Kung logic was developed in^14^. The Brent-Kung adder’s bigger silicon surface is a major disadvantage, though, and it may provide problems in applications with restricted space.

As highlighted earlier, the design of energy-efficient MAC units has garnered significant attention due to their critical role in applications such as DSP, machine learning, AI, and IoT-based edge devices. Many researchers have made several contributions to the advancement of MAC, most of which are centered around reducing the power consumption and area while increasing the throughput and scalability. An innovative MAC unit design^17^ has been introduced which incorporates approximate multipliers, intentionally trading off some computational accuracy to achieve significant power consumption reductions. This design proves especially beneficial for error-tolerant applications, such as image and video processing, where slight accuracy deviations are acceptable. Their work emphasizes the potential of approximate computing to address power constraints in modern processors. The research work^18^ proposes the use of up to six approximate MAC (AMAC) units to maximize performance efficiency while minimizing the error rate. This approach resulted in a remarkable 64% reduction in area and power compared to conventional MAC units in image smoothing applications. An energy-efficient MAC design has been presented^19^ using approximation computing, where errors are compensated during the accumulation phase by interleaving various approximate multipliers. This method enhanced energy efficiency by 35% without compromising recognition accuracy.

The study presented in^20^ integrates the Karatsuba multiplication algorithm with Wallace tree multipliers^21^ to design an energy-efficient multiply-accumulate (MAC) unit. By incorporating the computational efficiency of Karatsuba’s algorithm and the parallel processing capability of Wallace trees, the design achieves a balance between high performance and low energy consumption. The proposed Karatsuba double MAC architecture increases the efficiency of up to 80% for various CNN applications. The research work^22^ proposes a new strategy for multiplication of two numbers with interlaced partition multiplication, then follows the traditional addition operation in a MAC unit. This approach has only shown marginal speed improvement when compared to traditional architecture. A bit-serial computation MAC^23^ called the bitMAC has been proposed. This architecture has shown considerable improvement in reduction in area, power efficiency, and latency compared to conventional models and saves 52.79% of leakage power. A power-efficient and reduced-area MAC design^24^ that combines modified Booth multipliers and a 4-bit adder in combination with a 28-bit look-up adder is used as a hybrid adder. To improve the performance, a pipeline is introduced in the multiplier and accumulate section by breaking them into stages. There was a good throughput improvement in this approach, and it is suitable for resource-constrained applications. For real-time embedded applications, a pre-computation-based multiplexer network multiplier for MAC units^25^ has been proposed. It has shown a remarkable 13% reduction in area and consumes 35% less power than standard booth multipliers. Pipelined MAC units improve performance by shortening critical paths. Selective elimination of flip-flops, especially in machine learning accelerators, can save 20% in energy and area without violating functional requirements^26^.

Flexible MAC units^27^ support both fixed-point and floating-point operations, with configurable bit-widths for exponent and mantissa has been proposed. This allows efficient support for a range of neural network precisions, from binary to 16-bit floating point, with modest area overhead. Floating point 16-bit pipelined MACs improve accuracy for neural network training but at the cost of higher area and power compared to fixed-point MACs^28^. Optimized designs can still achieve up to 93% power reduction compared to traditional floating-point MACs. FPGA-based MACs support both low-precision and floating-point operations, enabling flexible trade-offs between accuracy and efficiency for AI and DSP workloads, as explored in^29^. Quantum-based MAC units using Vedic multipliers and single-layer layouts introduced in^30^ achieve low latency and minimal area, suitable for future nanoscale digital signal processing. A flexible multi-precision MAC, which has a 16-bit multiplier, or a sum of two 8-bit multipliers and a 16-bit adder primarily designed to accommodate floating-point operations to suit DSP applications, was investigated by^31^. This flexible MAC shows a 21.8% area overhead and less hardware requirement compared to standard 16-bit and 32-bit MACs, respectively. A 32-bit MAC unit, for IoT processor, is proposed in^32,33^, which can perform three operations of 16-bit and 32-bit signed and unsigned numbers. This 32-bit MAC decreased the delay and consumes less power.

Despite significant advancements in MAC architecture optimization, existing systems largely lack automation and flexibility to meet diverse design requirements. The proposed approach addresses these limitations by introducing an adaptable framework for automated MAC unit generation tailored to application-specific needs. In this study, an automated MAC generation environment capable of dynamically producing synthesizable Verilog HDL for user-defined bit-widths is presented. The framework employs efficient design strategies such as carry-skip adders^34^ to minimize propagation delay and Dadda^35^ and Wallace tree multipliers for high-speed, parallel multiplication. By automating the design process and reducing manual intervention, the framework significantly shortens development time and enhances scalability across a wide range of application domains. This architecture also serves as a foundation for hardware accelerators targeting edge computing and next-generation AI applications^36,37^, where resource efficiency and adaptability are of primary importance.

The research not only overcomes the limitations of traditional MAC unit designs but also promotes further exploration into automated and reconfigurable hardware architectures optimized for emerging AI technologies. Furthermore, the proposed framework aligns with global efforts toward energy-efficient and application-specific hardware accelerators, enabling seamless integration into future AI and ML ecosystems.

The key contributions of this research work are as follows:

1. Development of a scalable and automated Python-based framework for generating synthesizable Verilog HDL code for MAC units.
2. Significant reduction in manual design effort and acceleration of prototyping for MAC components used in DSP and AI applications.
3. Comprehensive validation through functional simulation, RTL synthesis, and physical design using Cadence tools on Generic Process Design Kit (GPDK) 180 nm,90 nm and 45 nm technology nodes.
4. A 70.08% reduction in delay and a 91.2% improvement in Energy Delay Product (EDP) were achieved for the proposed 16-bit GD-based MAC architecture compared to existing designs synthesized at the 90 nm technology node. These results further confirm the scalability and energy efficiency of the proposed MAC architecture across advanced technology nodes.

The remainder of this paper is organized as follows: Sect. “Methodology” details the methodology employed for MAC design generation and validation. Section “ Results and Discussion” presents the experimental results and discussion of the proposed Python framework, including both front-end and back-end validation outcomes. Section 4 concludes the paper by summarizing the findings and outlining potential future directions.

## Methodology

This research presents a framework model that automatically generates HDL Verilog code for a MAC unit of any bit size, reducing manual design effort. It enhances scalability and adaptability for various computing applications. The proposed framework model is developed using a Python automation script. The automated MAC HDL Verilog code, configurable for any bit size, is simulated with Cadence^®^nclaunch, synthesized using Cadence^®^Genus, and its physical design is implemented using Cadence^®^Innovus tools using GPDK 180 nm,90 nm and 45 nm technology node libraries.

The core of MAC unit consists of GD multiplier^38^, carry skip adder^34^ and an accumulator. GD multiplier integrates both Wallace and Dadda multiplier algorithms. This hybrid design optimizes the trade-off between computational speed and area efficiency with parallel processing of the partial products. Following the multiplication stage, the addition process is performed using a carry-skip adder, which effectively reduces propagation delay by minimizing the impact of carry chains. The accumulation stage is implemented through a series of D flip-flops, ensuring reliable storage and transfer of partial sums across multiple clock cycles. This architectural approach enhances the overall efficiency and performance of the MAC unit in high-speed arithmetic operations.

### HDL verilog code generation

**Fig. 1:**
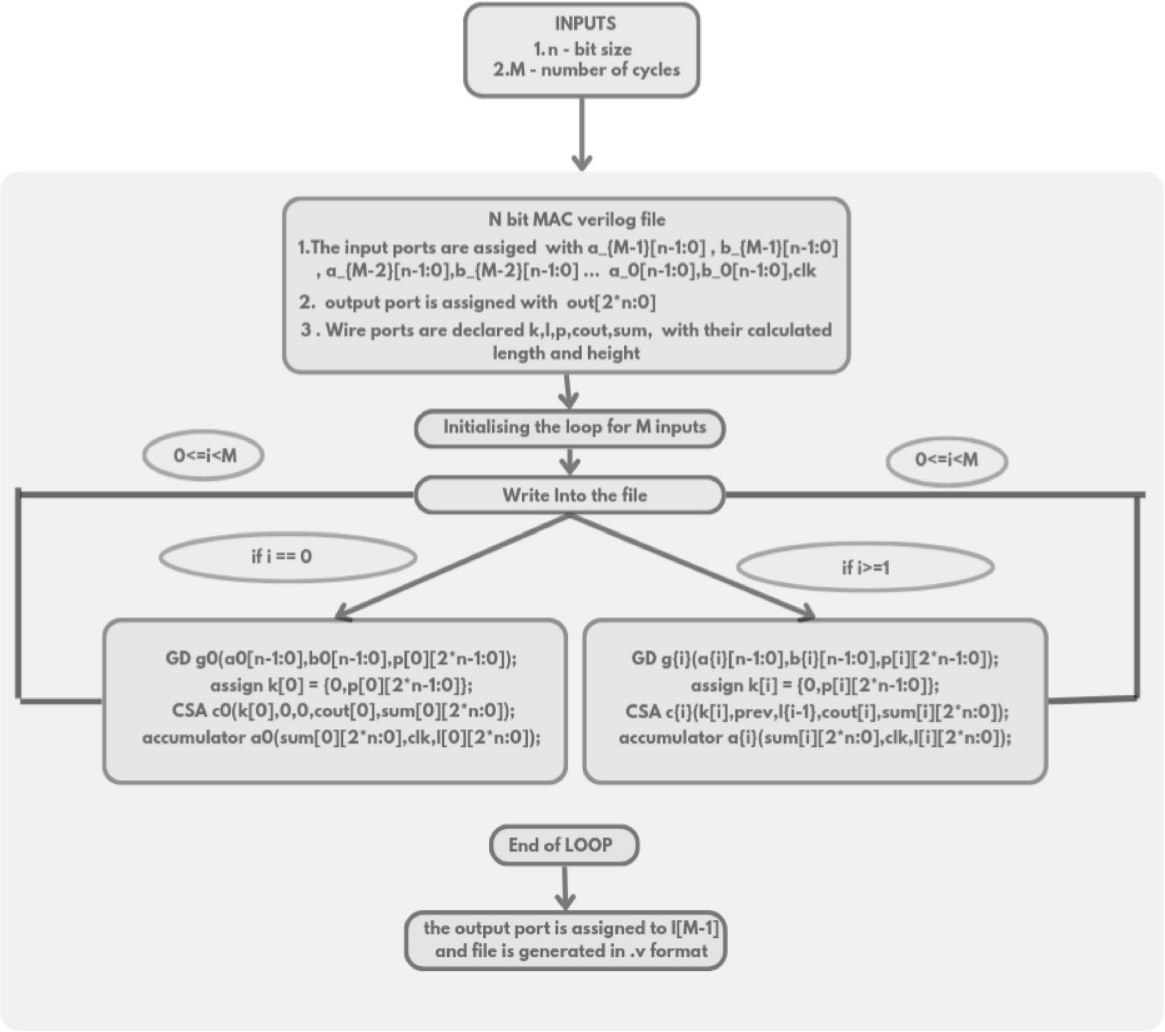
Flow diagram of n-bit MAC Verilog file generation.

**Fig. 2:**
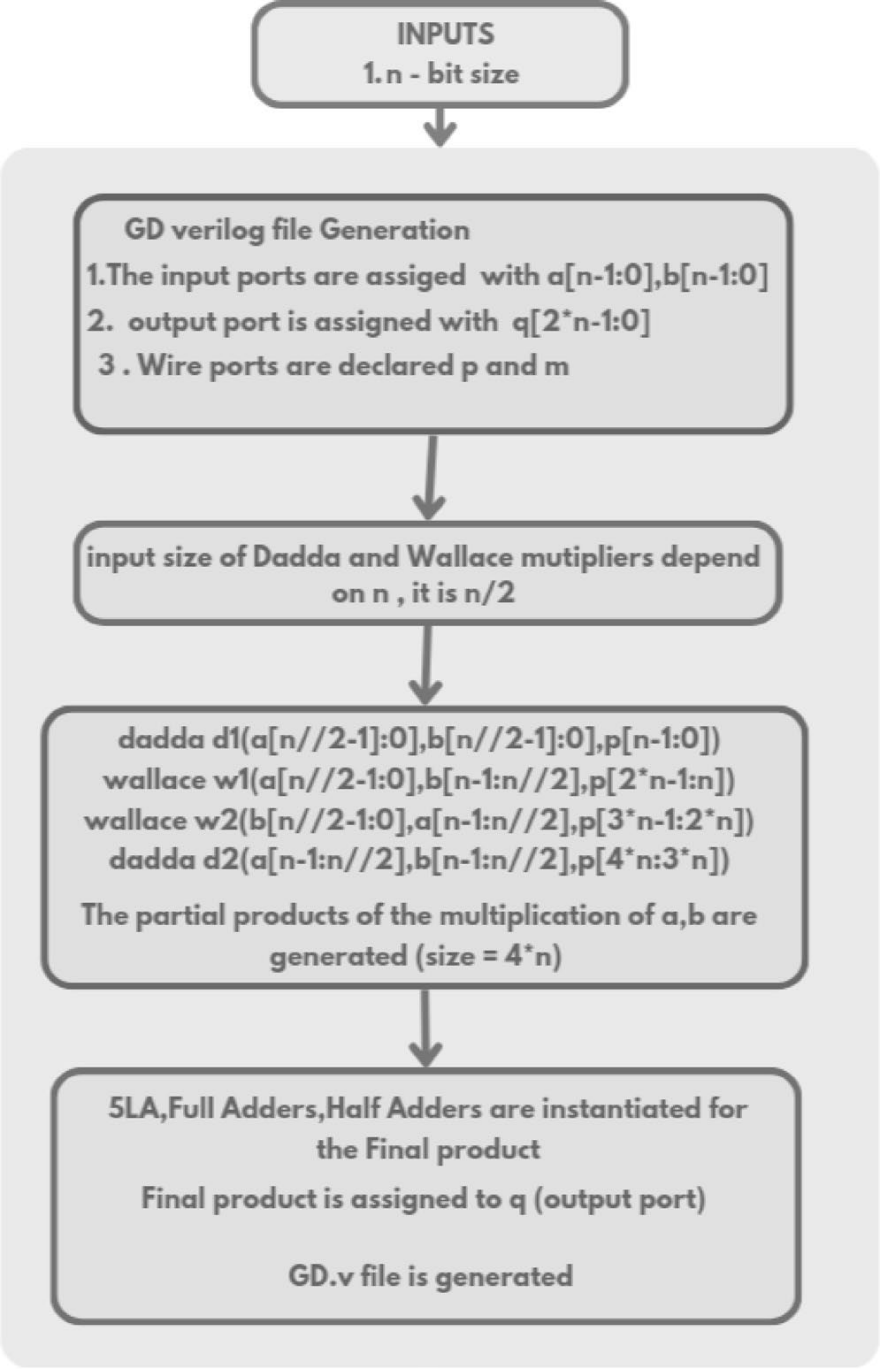
Flow diagram of n-bit GD multiplier Verilog file generation.

**Fig. 3:**
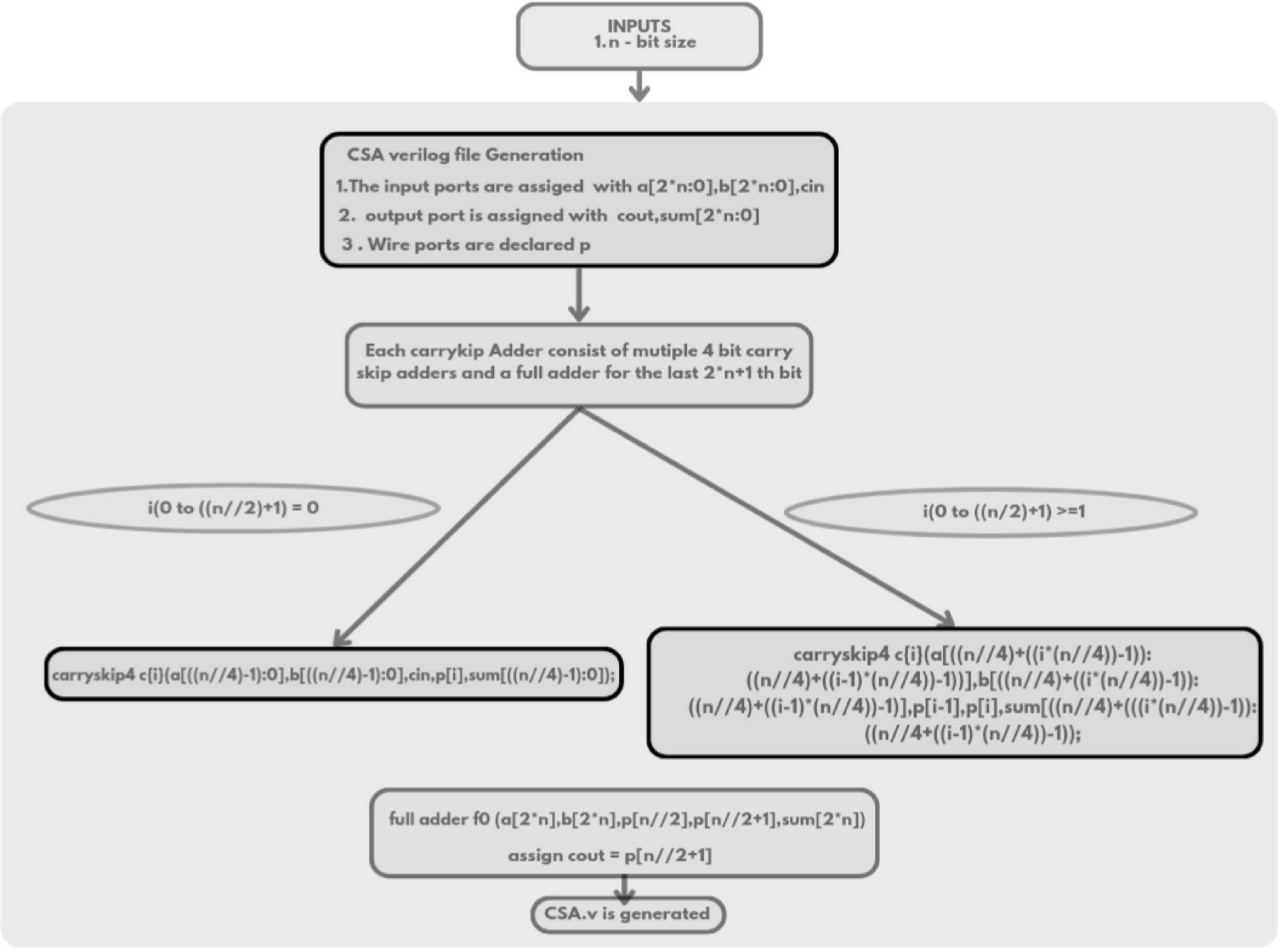
Flow diagram of (2n + 1)-bit carry skip adder Verilog file generation.

**Fig. 4:**
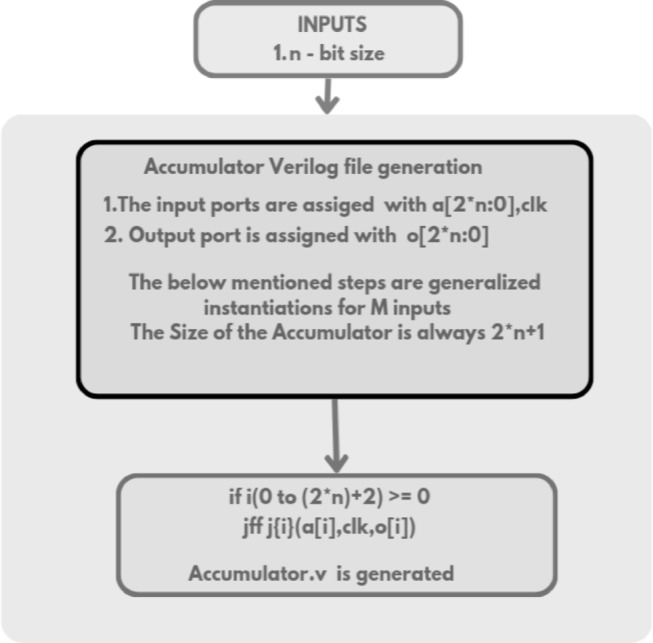
Flow diagram of (2n + 1)-bit accumulator Verilog file generation.

**Fig. 5:**
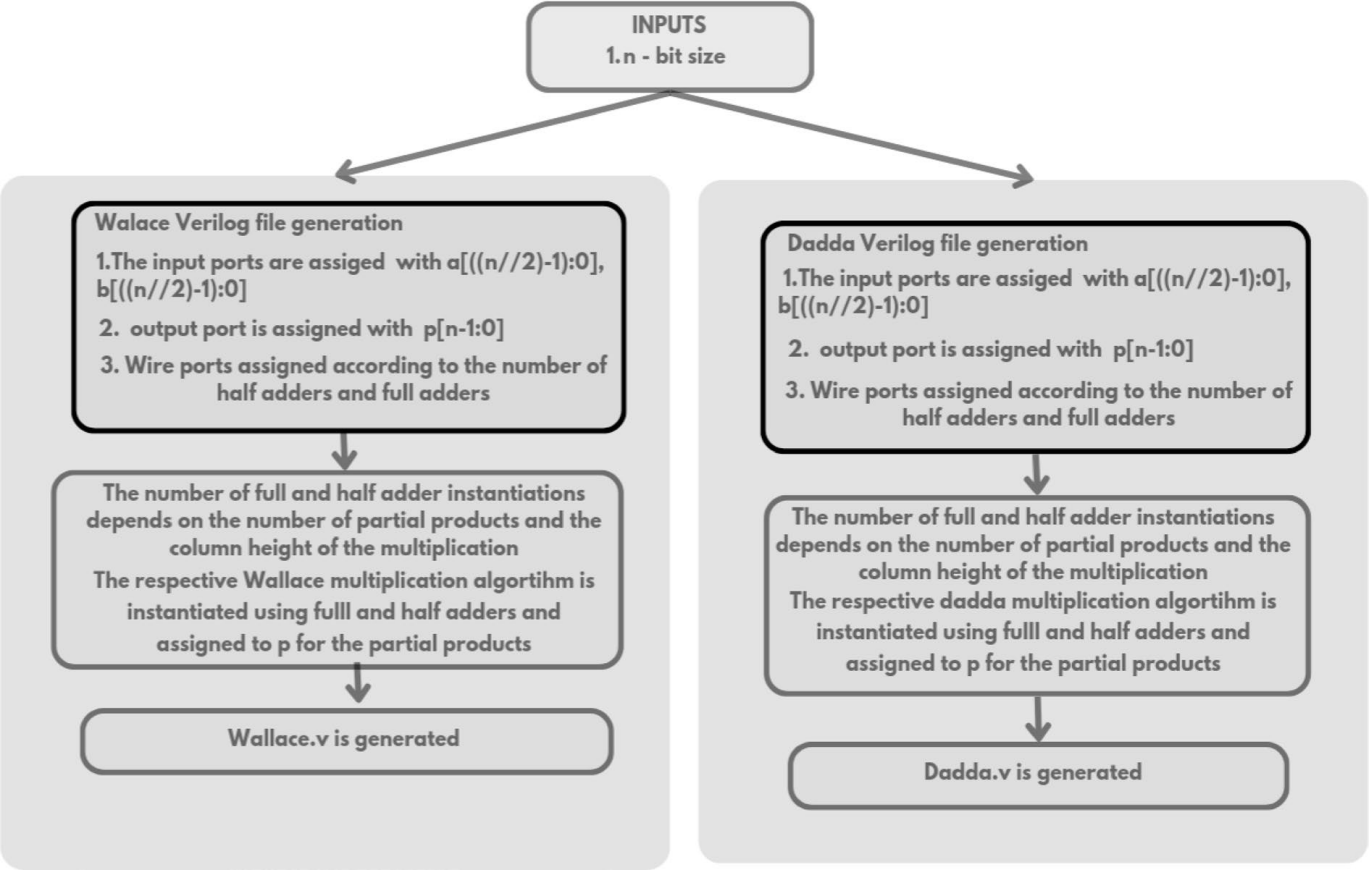
Flow diagram of n/2 Wallace and Dadda binary multipliers Verilog file generation.

The Verilog code for an n-bit MAC unit is automatically generated using the proposed Python script, based on the flow diagram shown in Fig. 1. The script takes two input parameters: n representing the bit size of the MAC unit, and M, denoting the number of input cycles to the MAC unit. The n-bit MAC consists of three primary submodules: an n-bit GD multiplier, a (2n + 1)-bit carry-skip adder, and a (2n + 1)-bit accumulator. The Verilog files for these submodules are generated concurrently, as illustrated in the flow diagrams shown in Figs. 2 and 3, and 4, respectively. The n-bit GD multiplier itself is composed of two types of binary multipliers: n/2 Wallace multipliers and n/2 Dadda multipliers. The flow diagram depicting the Verilog file generation process for these binary multipliers is shown in Fig. 5.

The pseudocode presented in Tables 1, 2, 3, 4, 5 and 6 outlines the workflow of the proposed Python script for automatically generating Verilog files required for a 16-bit MAC design, based on the corresponding flow diagrams with M = 3 input cycles. The process begins by generating the top-level module **mac.v** as shown in Table 1 which declares input ports for three pairs of 16-bit operands (a_0 to a_2 and b_0 to b_2), a clock signal (clk), and a 33-bit output (out). For each input cycle i (ranging from 0 to 2), the script creates three submodules— GD multiplier (***GD.v***), carry skip adder (***carryskip.v***), and an accumulator (***accumulator.v***) which are instantiated within the ***mac.v*** top module.

The ***GD.v*** HDL module has been developed to perform 16-bit multiplication as part of a 16-bit MAC unit with M = 3 input cycles, by decomposing the operation into four 8 × 8-bit segments, as illustrated in Table 2. In each cycle, the ***GD.v*** module computes a 32-bit partial product Q[i] from the corresponding 16-bit inputs a[i] and b[i]. This product is then extended by padding a leading zero to form a 33-bit intermediate result K[i]. To efficiently reduce the partial products, the GD multiplier incorporates two set of 8-bit submodules: ***dadda.v*** and ***wallace.v***, which implement the Dadda and Wallace tree multiplication algorithms, respectively. These submodules use arrays of full adders and half adders to optimize the height and depth of the partial product reduction tree, as described in Tables 3 and 4. During the final reduction stage, the ***GD.v*** module utilizes 5:3 compressor circuits in combination with full adders and half adders to arrive the final 32-bit product, ensuring high speed and logic efficiency. This functionality is realized through the automatic generation of supporting Verilog files—***la.v***,*** halfadder.v and fulladder.v***—tailored to meet the specific bit-width requirements of the design.

The ***carryskip.v*** HDL module has been generated to perform 33-bit addition within a 16-bit MAC unit, as detailed in Table 5. This addition is implemented using the carry skip algorithm, which divides the input operands into 4-bit blocks to enable efficient carry propagation across the adder. A series of ***carryskip4.v*** submodule which includes mux.v submodule generation to select the carry signal and to handle these blocks, while a dedicated full adder manages the most significant bit to minimize the delay. In the initial cycle (i = 0), the carry skip adder adds K[0] with two zero-valued operands, producing sum and carry outputs that are stored in a 33-bit accumulator implemented with edge-triggered flip-flops. For subsequent cycles (i ≥ 1), the adder processes K[i], the previous accumulator output l[i-1], and the carry output cout[i] from the prior stage. The resulting sum and carry signals are again captured in the accumulator.

Accumulation across each cycles is handled by the ***accumulator.v*** submodule. The HDL code of 33-bit accumulator.v which is required for 16-bit MAC is generated by instantiating a flip-flop for each of the 33 bits through ***fliflop.v*** Verilog file generation, enabling pipelined operation as depicted in Table 6. The proposed Python script generates the following Verilog files concurrently as its final output, supporting any specified bit-width for the MAC unit.

- ***mac.v*** – This is the top-level Verilog module for the MAC unit. It instantiates the GD multiplier, carry-skip adder, and accumulator as its subsystems.
- ***GD.v*** – Contains the Verilog code for the GD multiplier, which instantiates the 5:3 compressor, full adder, half adder, Dadda multiplier, and Wallace multiplier as its subsystems.
- ***wallace.v*** – Provides the Verilog code for the Wallace multiplier, used as a subsystem within the GD multiplier as required.
- ***dadda.v*** – Contains the Verilog code for the Dadda multiplier, used as a subsystem within the GD multiplier as required.
- ***la.v*** – Includes the Verilog code for the 5:3 compressor, which is used multiple times within the GD multiplier during the final reduction stage.
- ***halfadder.v*** – Provides the Verilog code for a single-bit half adder, used multiple times as a subsystem within the GD multiplier during the final reduction stage.
- ***fulladder.v*** – Contains the Verilog code for a single-bit full adder, used multiple times as a subsystem within the GD multiplier during the final reduction stage.
- ***mux.v*** – Includes the Verilog code for a 2:1 multiplexer, used as a subsystem inside the carry-skip adder to select the output corresponding to either Cin = 0 or Cin = 1.
- ***carryskip.v*** – Contains the Verilog code for the carry-skip adder, used as a subsystem within the MAC unit.
- ***carryskip4.v*** – Provides the Verilog code for a 4-bit carry-skip adder, used as a subsystem within the main carry-skip adder of the MAC unit.
- ***flipflop.v*** – Includes the Verilog code for a single-bit D flip-flop, used as a subsystem within the accumulator of the MAC unit.
- ***accumulator.v*** – Contains the Verilog code for the accumulator, used as a subsystem within the MAC unit.

**Table 1:**
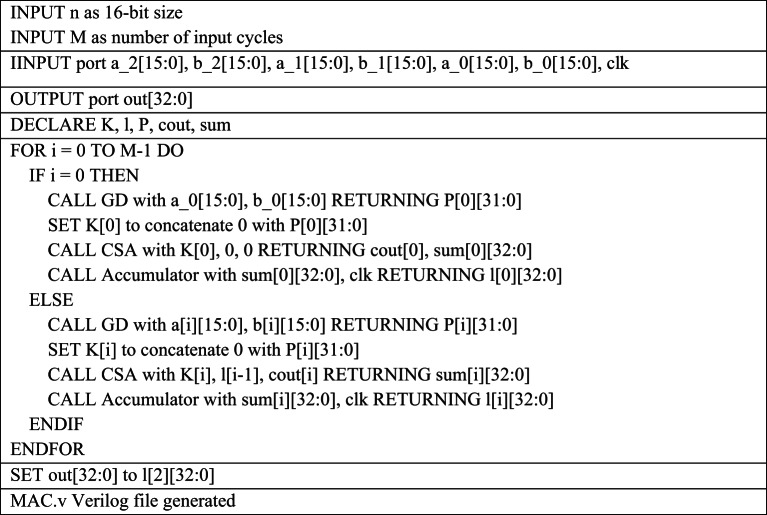
Pseudocode for 16-bit MAC Verilog file generation with M=3

The Python script developed to implement the pseudocode for MAC unit Verilog code generation (as shown in Table 1) begins by defining the MAC size and the number of input cycles as parameters. It then initializes list data structures to store signal names like P[i], K[i], sum[i], and l[i]. Using Python’s looping and string formatting capabilities, the script iteratively generates Verilog module instantiations and assignment statements for each input cycle. Conditional if–else logic is applied to distinguish the first iteration (i = 0), where zero initialization is required, from subsequent cycles that reuse previously generated signals. Each module call is formatted as a Verilog instance string and appended to a cumulative text buffer. Function blocks are employed to encapsulate repetitive patterns such as module headers, signal declarations, and port mappings, thereby improving readability and reusability. Finally, Python’s file I/O operations are utilized to write the complete Verilog code into the output file *mac.v.*

**Table 2:**
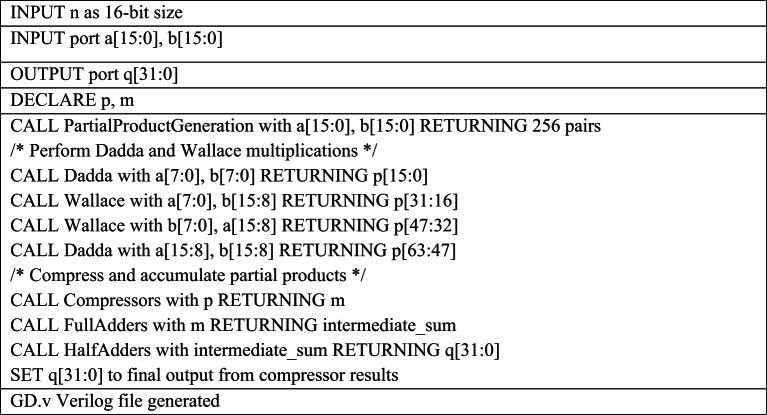
Pseudocode for 16-bit GD Multiplier Verilog file generation required for 16-bit MAC Design.

The Python script automates the pseudocode presented in Table 2 to generate the Verilog code for the GD multiplier in three structured phases. First, it uses variable slicing and string formatting to divide the 16-bit inputs into upper and lower halves and dynamically instantiate four multiplier modules—two Dadda and two Wallace—while appending their outputs to a shared internal bus. Next, the script programmatically generates *assign* statements to map the least significant bits from the first multiplier to the output. Finally, through iterative loops and formatted templates, it constructs a pipelined adder chain by instantiating full adders, compressors, and half adders in sequence. All generated Verilog lines are accumulated in a text buffer and written to the output file named *GD.v.*

**Table 3:**
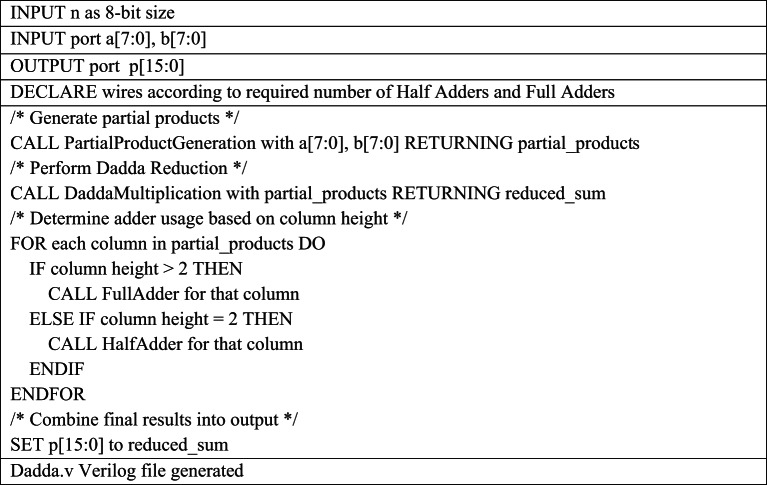
Pseudocode for 8-bit Dadda Verilog file generation required for 16-bit MAC Design.

The Python script for Dadda multiplier Verilog generation operates in three main phases as shown in Table 3. First, it generates the initial partial product matrix by performing bitwise AND operations on the multiplicand and multiplier bits and organizes the results into a columnar “dot diagram” structure, with each list representing a column of the same bit weight. Next, it performs iterative column reduction based on the Dadda sequence, automatically instantiating full adders and half adders to reduce columns exceeding the stage height limit, while managing intermediate sum and carry signals. Finally, after each column is reduced to two bits, the script generates a final two-input adder to compute the final product. All Verilog instances and signal connections are dynamically created and written to the output file named *dadda.v.*

**Table 4:**
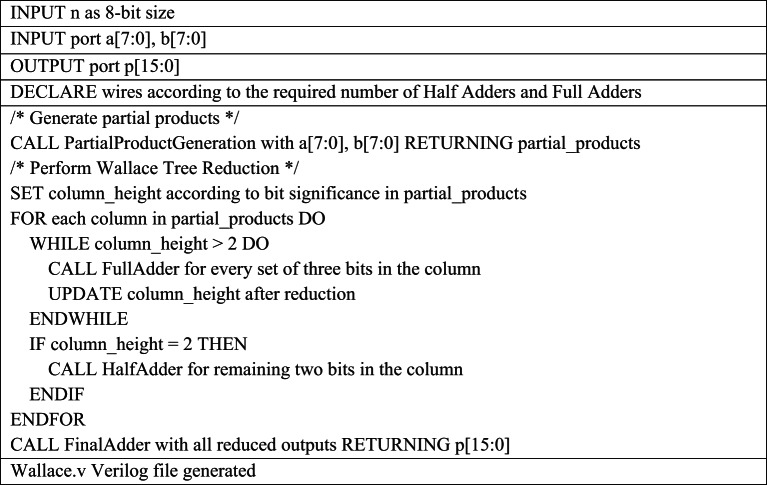
Pseudocode for 8-bit Wallace Verilog file generation required for 16-bit MAC Design .

The Python script for *wallace.v* generation automates the process in three main phases as shown in Table 5. It first programmatically generates the 8 × 8 partial product matrix for 16bit MAC unit design by iterating over input bits and performing bitwise ANDs, then organizes the results into a columnar data structure for easier manipulation. In the Wallace reduction phase, the script loops over each column, dynamically checking the column height and instantiating full adders (3:2 compressors) or half adders (2:2 compressors) as needed, updating the column and next-column bit lists after each reduction. The script uses formatted strings and template functions to generate the Verilog module instances and signal connections automatically. Finally, for the last two-row summation, it iteratively creates a ripple-carry adder by instantiating full and half adders from LSB to MSB, propagating carries programmatically and writing all generated Verilog lines into a text buffer, which is then exported to the output file *wallace.v*.

**Table 5:**
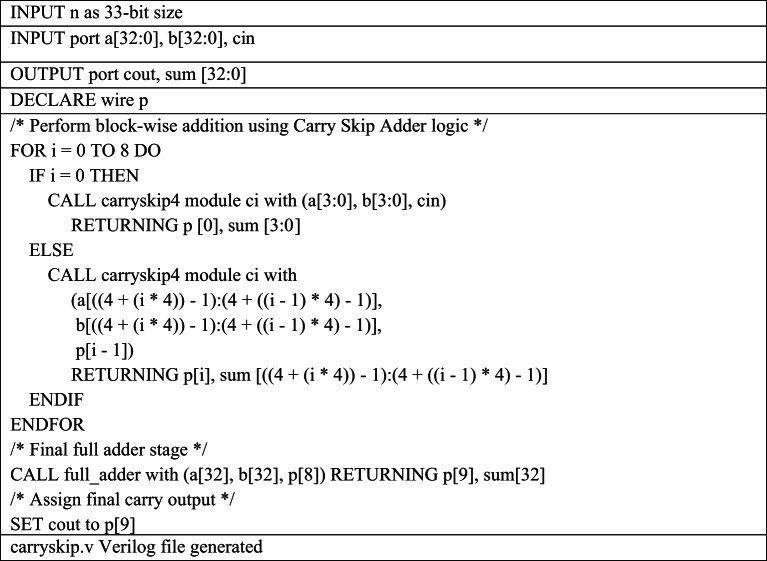
Pseudocode for 33-bit Carry Skip Adder Verilog file generation required for 16-bit MAC Design

The Python script for carryskip.v generation automates the construction of a 33-bit carry-skip adder in three phases as shown in Table 6. In the first phase, it calculates the start and end indices for each 4-bit block based on the total adder width, storing them in a boundary list to define bit slices and internal carry wires. In the second phase, it iterates over this boundary list and dynamically instantiates the appropriate Verilog module for each block: the LSB block connects its carry-in to the primary input and its carry-out to the first internal wire, while the middle blocks connect each carry-in to the previous block’s carry-out and each carry-out to the next internal wire, forming a chained carry-skip structure. In the third phase, the MSB block is implemented using a full adder, connecting its carry-in to the last skip-wire and assigning its carry-out to the module’s main output. Loops, conditional logic, and formatted strings are used throughout to generate Verilog instances and signal connections, which are accumulated in a text buffer and written to the carryskip.v file.

**Table 6:**
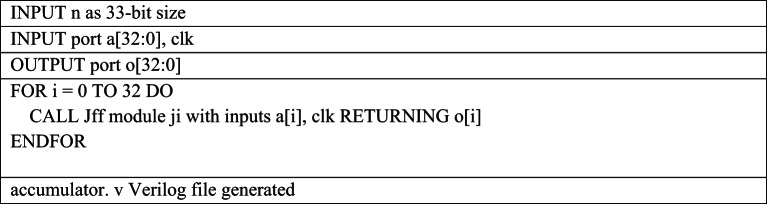
Pseudocode for 33bit Accumulator Verilog file generation required for 16-bit MAC Design

The Python script for accumulator.v automates the generation of a 33-bit D flip-flop based accumulator for 16-bit MAC unit design as shown in Table 6. It iterates over the 33 input bits using a loop, and for each bit, it dynamically instantiates a Jff module by formatting the module instance string with the corresponding input bit a[i], the clock signal clk, and the output bit o[i]. All generated Verilog instance lines are appended to a text buffer, ensuring proper naming and connectivity for each flip-flop. After completing the loop, the script writes the accumulated Verilog code into the output file accumulator.v.

### Internal architecture of GD multiplier, carry skip adder and an accumulator unit used in 16bit MAC

The Verilog HDL file for the MAC unit, generated by the proposed Python script, incorporates the GD multiplier, carry skip adder, and an accumulator algorithm for its internal operations. This section presents the internal architecture of these components, using a 16-bit MAC unit design as a representative example.

**Fig. 6:**
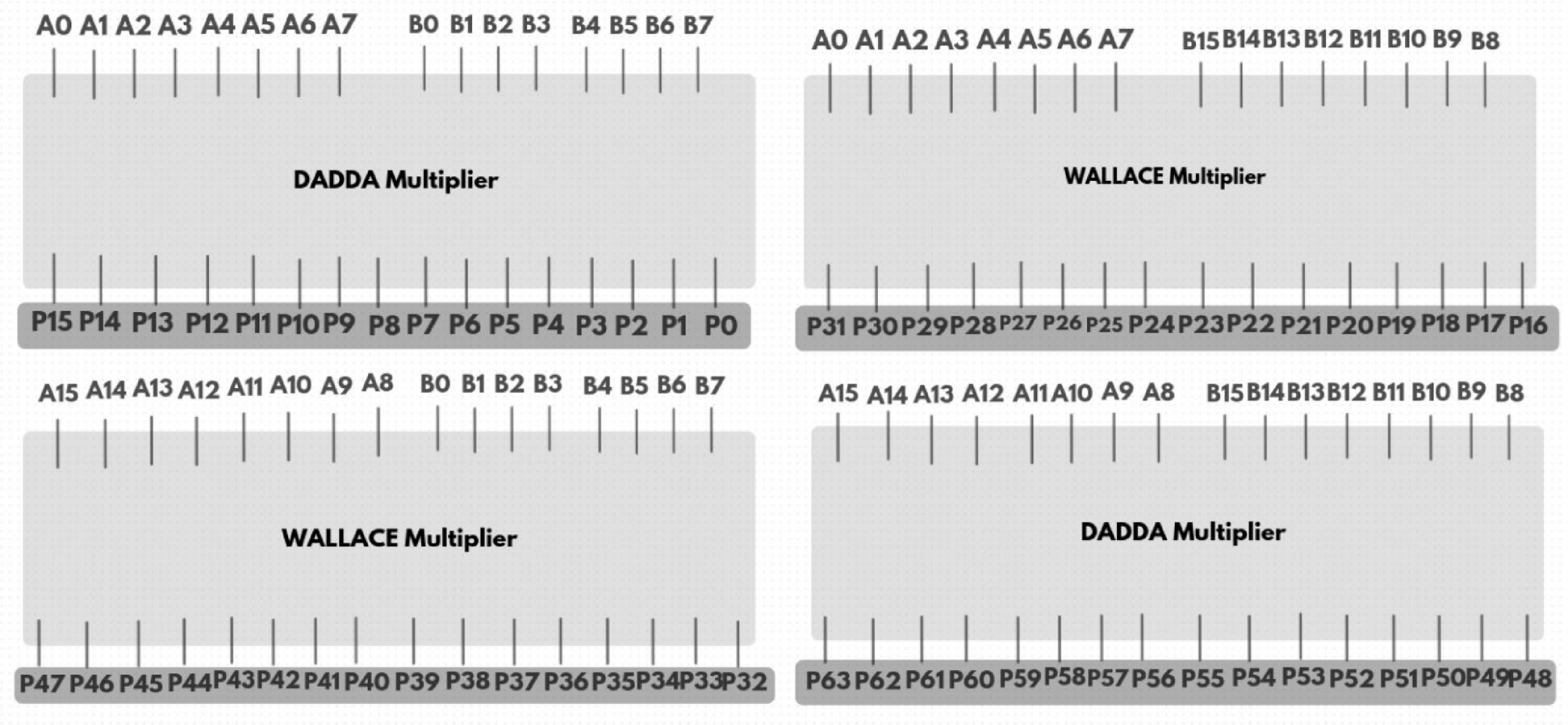
Grouping and decomposition procedure.

**Fig. 7:**
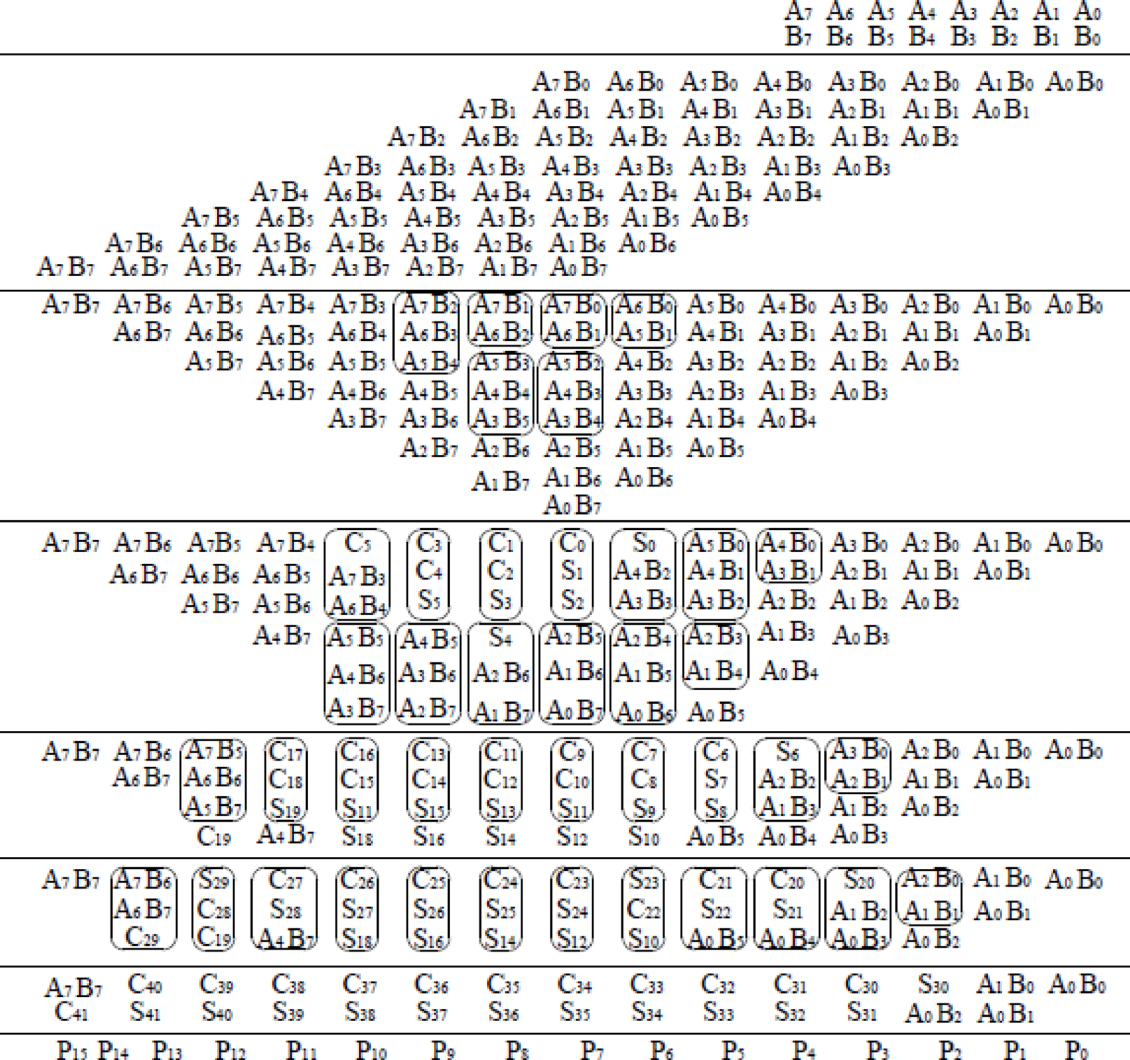
8-bit dadda multiplier^35^.

**Fig. 8:**
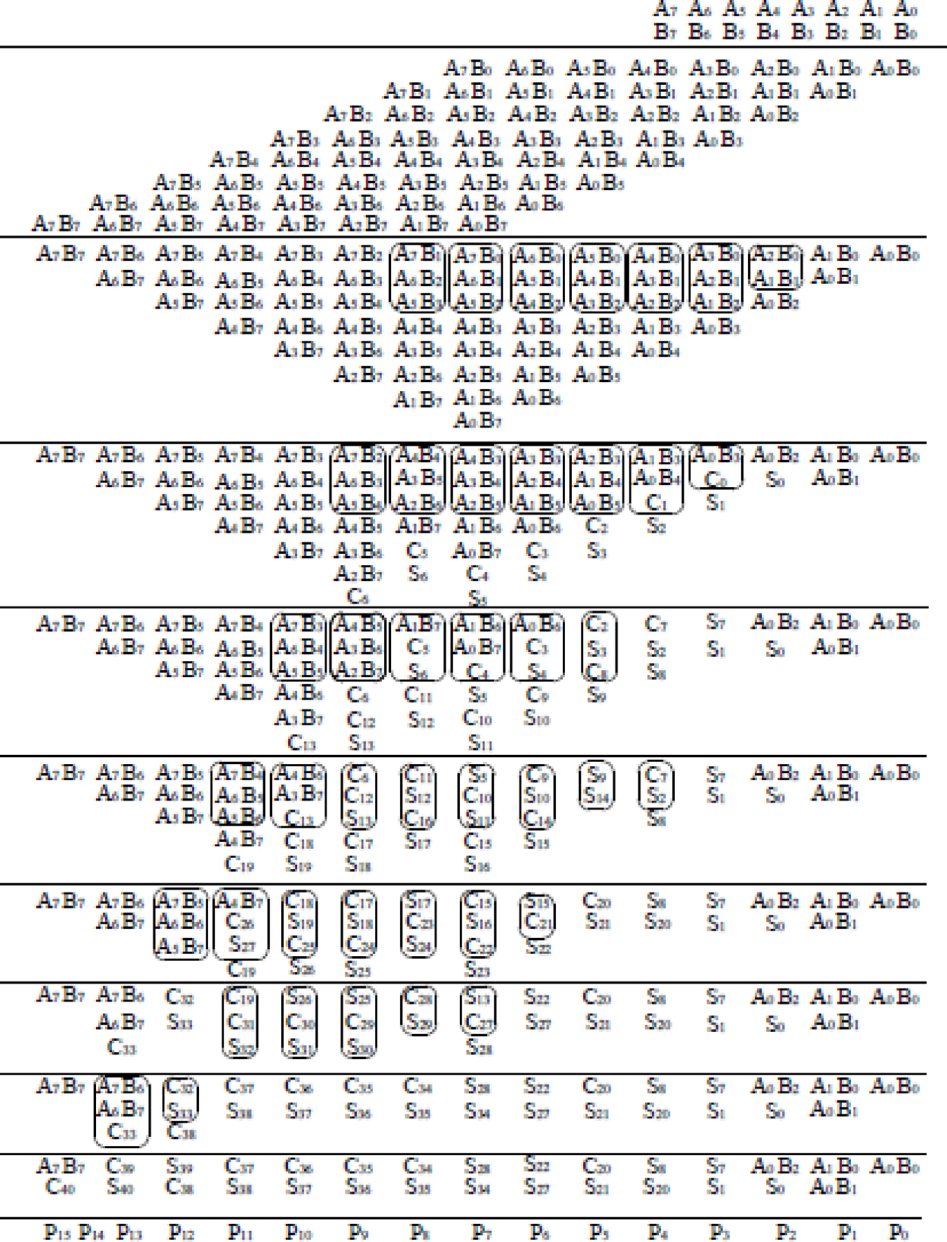
8-bit wallace multiplier^21^.

The GD Multiplier is a binary multiplier that begins by dividing the input operands into four separate groups. Each group performs multiplication in parallel using two sets of Wallace and Dadda tree algorithms. Figure 6 shows the grouping and decomposition procedure of 16-bit GD multiplier. The illustrated 16-bit GD Multiplier divides the input operands A [15:0] and B [15:0] into four overlapping 8-bit groups, enabling high-speed parallel multiplication. The top-left block is a Dadda multiplier that receives the least significant 8 bits of both inputs (A [7:0], B [7:0]) and generates partial products P0 to P15. The top-right block, a Wallace multiplier, processes A [7:0] and the most significant bits of B [15:8], outputting P16 to P31. Meanwhile, the bottom-left Wallace multiplier handles A [15:8] and B [7:0], producing P32 to P47. The bottom-right Dadda multiplier takes the most significant bits of both inputs (A [15:8], B [15:8]) and outputs P48 to P63. All four multipliers operate concurrently, significantly enhancing throughput. The resulting partial products from all blocks (P0–P63) are fed into a final summation stage comprising half adders, full adders, and 5:3 compressor circuits, which efficiently combine these results into the final multiplication output. This modular and parallel architecture reduces computation time and is well-suited for high-performance digital systems. The 8-bit Dadda and Wallace multiplier working principle is shown in Figs. 7 and 8 respectively.

The Dadda multiplier architecture for an 8-bit binary multiplication is designed to accelerate the multiplication process by systematically compressing the 64 generated partial products into a product result using a minimum number of logic stages. Initially, the partial products from the inputs A and B are arranged in an 8 × 8 matrix. The compression is guided by the Dadda sequence, a series of maximum allowed column heights defined in reverse starting from 2, which for 8-bit inputs becomes {2, 3, 4, 6}. The matrix is reduced through several stages using (3,2) counters (full adders) and (2,2) counters (half adders), ensuring that the height of each column does not exceed the target value at each stage. This reduction begins by compressing the matrix to a height of 6, then to 4, then to 3, and finally down to 2 rows. Once only two rows remain, a final carry-propagate adder combines them to produce the 16-bit product. This structured reduction approach, which delays the use of adders until necessary, minimizes both delay and hardware complexity, making the Dadda multiplier an efficient solution for high-speed binary multiplication.

The Wallace multiplier for 8-bit binary multiplication is a high-speed arithmetic circuit that reduces the delay associated with summing partial products by aggressively compressing them through parallel reductions. For two 8-bit inputs A and B, the multiplication generates 64 partial products arranged in an 8 × 8 matrix, with each bit of A multiplied with each bit of B. The Wallace tree reduction begins by immediately applying full adders (3,2 counters) and half adders (2,2 counters) to columns containing three or more bits, aiming to reduce the height of each column as rapidly as possible, typically cutting the number of bits in a column by approximately half in each stage. This approach continues until the matrix is reduced to just two rows, which are then passed into a conventional carry-propagate adder to produce the final 16-bit product. Parallelism and early reductions significantly decrease propagation delay.

**Fig. 9:**
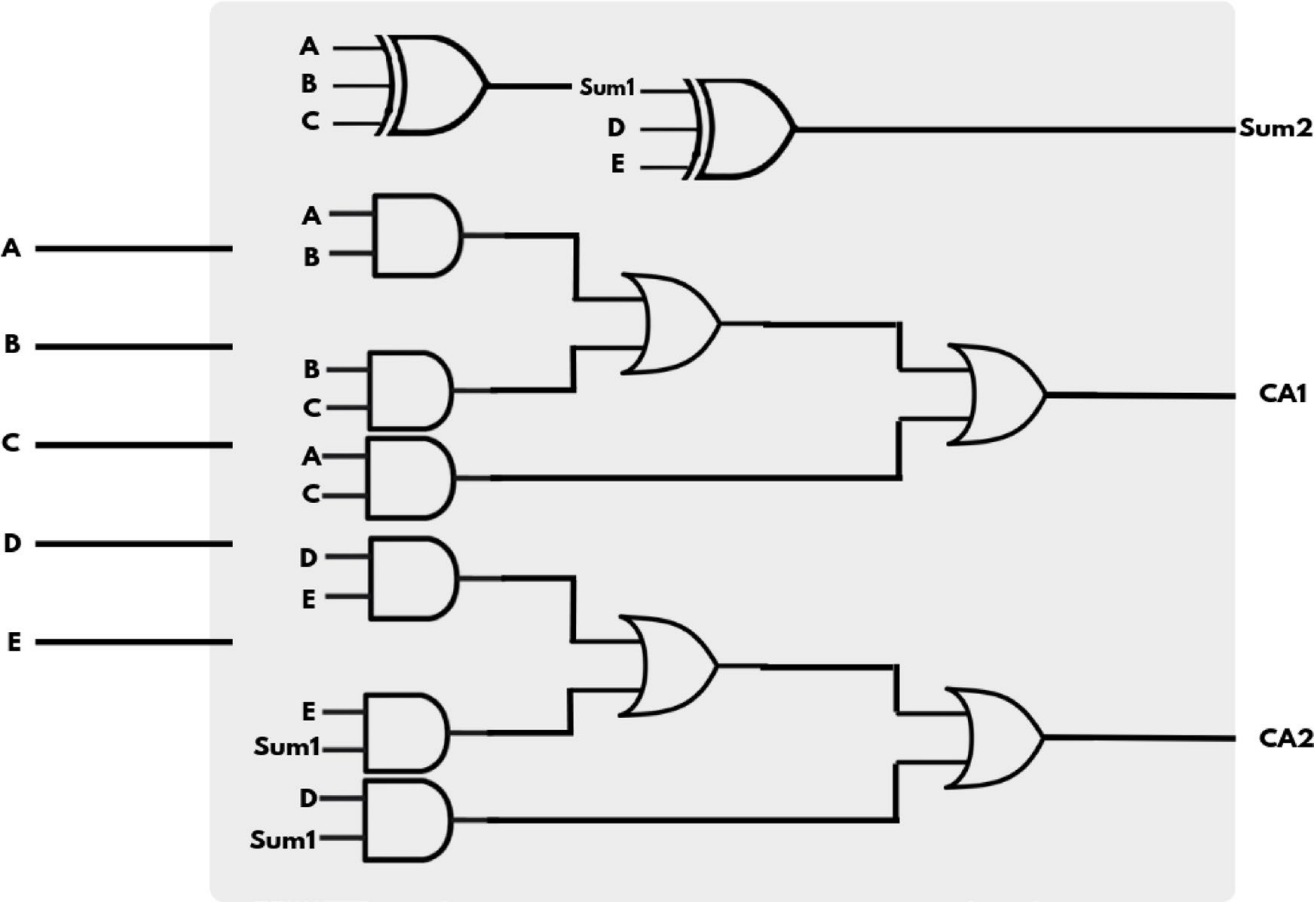
5:3 Compressor logic diagram[5LA].

Figure 9 illustrates the internal architecture of a 5:3 compressor, a key component of the GD multiplier employed within the MAC unit. This design effectively compresses five input bits (A, B, C, D, E) into three outputs: two carry outputs (CA1, CA2) and a final sum output (SUM2). The compression is achieved using two cascaded full adders. In the first stage, inputs A, B, and C are processed by the first full adder to produce an intermediate sum (SUM1) and a carry (CA1). In the second stage, this intermediate sum (SUM1) is fed into a second full adder along with inputs D and E, resulting in a final sum (SUM2) and another carry output (CA2). This structure efficiently reduces the number of partial product bits during multiplication while maintaining fast processing speeds by minimizing the depth of the carry propagation path. The dual carry outputs allow for greater flexibility in routing intermediate results to subsequent compression stages, making the 5:3 compressor a highly efficient building block in advanced digital multipliers.

The 16-bit GD multiplier^38^ integrates the outputs of Wallace and Dadda multipliers, which are efficiently combined using compressor, full-adder, and half-adder circuits to obtain the final product. Architecture employs a 33-bit carry-skip adder^34^, organized into multiple 4-bit blocks with skip logic to minimize propagation delay and enhance computational speed. An accumulator comprising 33 D flip-flops^5^ enables reliable storage of intermediate results, ensuring synchronized and stable operation across clock cycles for high-performance MAC functionality.

## Results and discussion

This section provides a detailed analysis of the proposed automated HDL generation framework for the design of Multiply-Accumulate (MAC) units with reconfigurable bit-widths, developed using a Python-based automation script. The framework’s efficacy is validated through the generation of both 8-bit and 16-bit MAC units, demonstrating its scalability and adaptability. The resulting designs functional checking were done through simulation, synthesized to generate RTL netlist(using GPDK 180 nm, 90 nm and 45 nm technology node libraries) and physical design implementation were performed by using GPDK 180 nm and 90 nm technology node libraries. The analysis highlights the framework’s ability to significantly reduce manual design effort across varying computational requirements. Furthermore, the selection of the GD multiplier, carry-skip adder, and accumulation algorithms in the MAC design facilitates parallelism, thereby improving computational speed. A comparative analysis with existing approaches is presented, demonstrating the performance benefits achieved through the proposed methodology.

### Automatic HDL code generation for n bit MAC

The proposed Python-based automation script enables automated generation of Verilog HDL code for an n-bit Multiply–Accumulate (MAC) unit, configurable for a user-defined number of inputs and computation cycles. The script employs advanced list manipulation techniques including list comprehension, recursive traversal, diagonal extraction, and column-wise restructuring to construct multi-dimensional data representations that model the hardware architecture. By combining mathematical sequence generation with algorithmic filtering, the framework synthesizes partial product arrays and reduction stages, and generates HDL code for various MAC unit modules, such as Wallace and Dadda tree multipliers, carry-skip adders, and accumulator logic. Each module is produced through structured string manipulation of predefined architectural templates, ensuring consistency and scalability in code development.

**Fig. 10:**
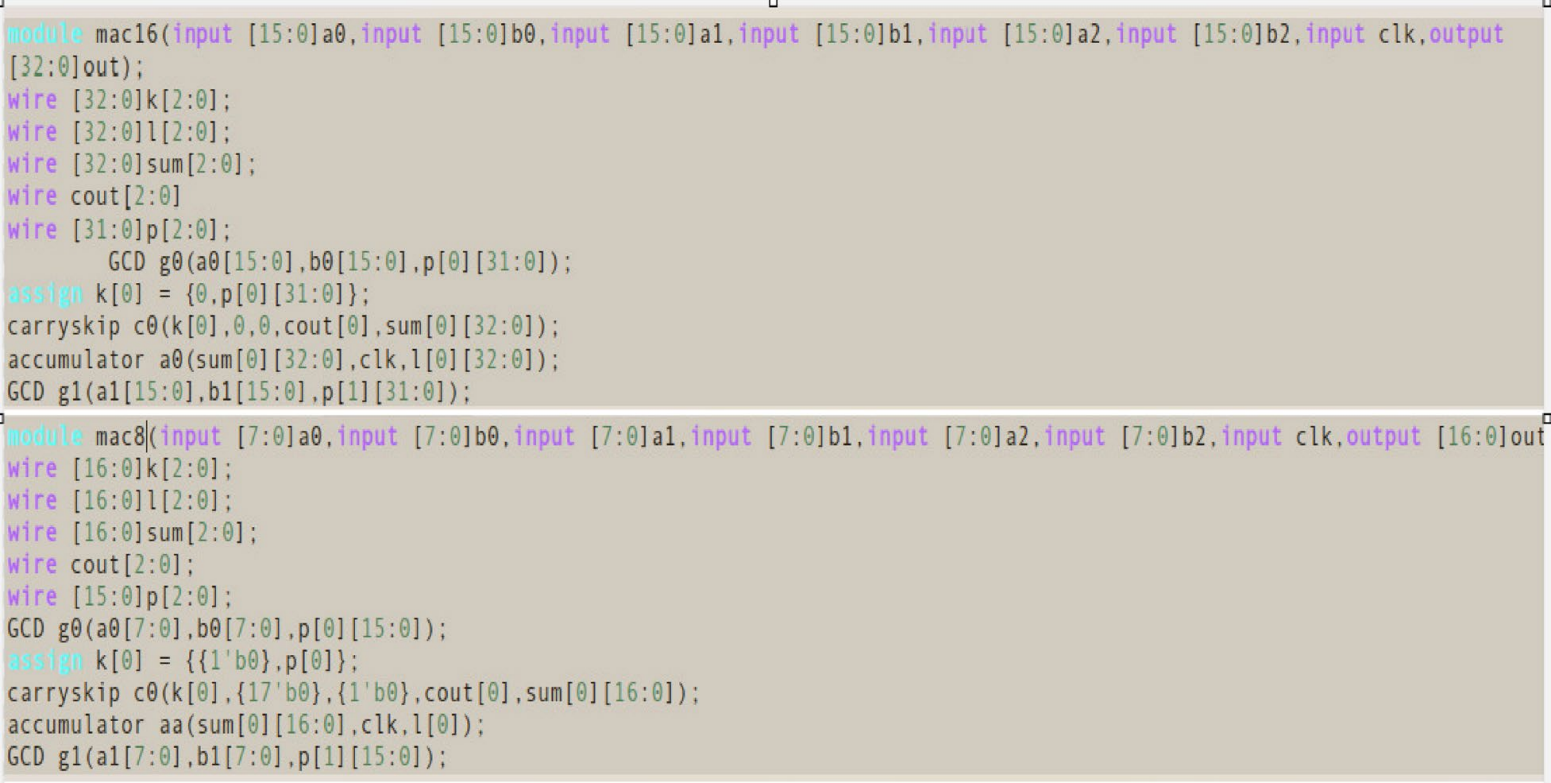
Comparative view of the top-level Verilog modules of 16-bit MAC and 8-bit MAC.

Figure 10 presents a comparative view of the top-level Verilog modules of 16-bit MAC and 8-bit MAC, generated by the proposed Python-based HDL generation script. The figure demonstrates the proposed framework model scalability and configurability across varying operand bit-widths and input sizes. The upper portion of the figure depicts the mac16 module generated for a 16-bit MAC configuration, wherein all input operands *(a0–a2*,* b0–b2)* are 16 bits wide, and the output operand *out* is 32 bits to accommodate accumulated product results. Module instantiations including carry skip adder, accumulator, and GD multiplier units are parameterized accordingly. In contrast, the lower portion of the figure illustrates the mac8 module generated for an 8-bit MAC configuration. The input operands are 8 bits wide, and the output width is reduced to 16 bits. All internal signal declarations and module port connections are correspondingly scaled to reflect the reduced bit-width. Despite the variation in word length, the structure, naming convention, and logical sequencing remain consistent, showcasing the syntactic uniformity achieved through templated code generation. The correctness and integration of generated modules have been validated through simulation and synthesis, confirming the framework model utility in rapid prototyping of arithmetic-intensive digital designs. The generated modules are reusable and portable, promoting efficient IP integration across ASIC and FPGA platforms.

### Functionality verification and frontend design of 8-bit and 16-bit MAC unit

**Fig. 11:**
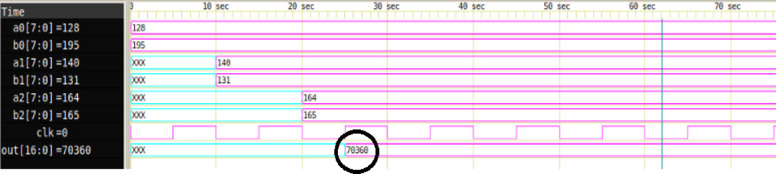
8-bit MAC simulation result with M = 3.

Figure 11 depicts the simulation waveform of an 8-bit MAC unit with three operand pairs (*M = 3*), generated using the proposed automated Verilog code generation framework. The inputs a0, a1, a2 and b0, b1, b2 represent the multiplicand and multiplier sets respectively, each 8 bits wide. As seen in the waveform, the input values are assigned as follows: a0 = 128, b0 = 195; a1 = 140, b1 = 131; and a2 = 164, b2 = 165. The MAC operation performs a parallel computation of partial products followed by accumulation, expressed as shown in equ 1. The result 70,360 appears on the out [16:0] output signal, confirming the correct functional behavior of the MAC unit. The simulation demonstrates proper synchronization through the clock (clk) signal and highlights the unit’s ability to handle multi-operand accumulation efficiently.

1 $$\:output=\left(a0\:\times\:b0\right)+\left(a1\:\times\:b1\right)+\left(a2\:\times\:b2\right)=\left(128\:\times\:195\right)+\left(140\:\times\:131\right)+\left(164\:\times\:165\right)=70360\:\:\:$$

**Fig. 12:**
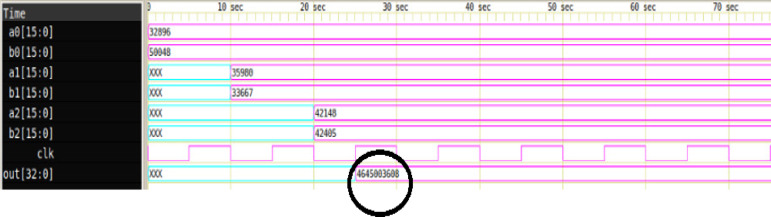
16-bit MAC simulation result with M = 3.

Figure 12 illustrates the simulation result of the proposed 16-bit Multiply-Accumulate (MAC) unit with three input operand pairs (*M = 3*), generated using the automated HDL code framework. The operands are assigned as follows: *a0 = 32,896*,* b0 = 50,048*,* a1 = 35,980*,* b1 = 33,667*, and *a2 = 42,148*,* b2 = 42,405*. The MAC operation performs a parallel computation of partial products followed by accumulation, expressed as shown in equ 2. The computed result is correctly reflected on the output signal *out[32:0].* The waveform validates the correct timing behavior and functional integrity of the MAC unit, as governed by the rising edge of the *clk* signal.

2 $$\:output=\left(a0\:\times\:b0\right)+\left(a1\:\times\:b1\right)+\left(a2\:\times\:b2\right)=\left(32896\times\:50048\right)+\left(35980\times\:33667\right)+\left(42148\times\:42405\right)=4645803668\:\:\:$$

To evaluate the scalability and performance adaptability of the MAC unit designs across different technology nodes, automated Verilog code for 8-bit and 16-bit MAC units was synthesized using the Cadence^®^ Genus tool with GPDK 180 nm, 90 nm, and 45 nm technology libraries. Table 7 presents a comparative analysis of key frontend design performance metrics, including propagation delay, power consumption, area utilization, and derived energy-performance parameters.

The results exhibit a clear trend of performance enhancement and efficiency improvement as the technology scales down. For the 8-bit MAC, the propagation delay reduces progressively from 9.758 ns (180 nm) to 8.412 ns (90 nm), and further to 1.12 ns (45 nm), demonstrating significant timing improvement due to faster transistor switching and reduced parasitic effects. Similarly, power consumption decreases from 1.412 mW to 0.339 mW, and sharply to 0.057 mW at 45 nm, marking an overall reduction of nearly 96%. The chip area also scales down substantially from 19865.261 μm² (180 nm) to 1702.22 μm² (45 nm) reflecting enhanced compactness and transistor density achieved with smaller geometries. Consequently, performance efficiency metrics such as PDP, EDP, and EOP show consistent improvements, reaching as low as 0.069 pJ, 0.072 pJ·ns, and 0.008 pJ/bit, respectively, at the 45 nm technology node.

A similar trend is observed in the 16-bit MAC design. The propagation delay improves from 17.738 ns (180 nm) to 11.094 ns (90 nm) and further to 2.282 ns (45 nm), while power consumption drops from 2.54 mW to 0.659 mW. The area utilization significantly reduces from 72355.479 μm² (180 nm) to 8418.84 μm² (45 nm). Correspondingly, the PDP improves from 45.05 pJ to 1.503 pJ, and the EDP from 799.17 pJ·ns to 3.432 pJ·ns, demonstrating the advantages of deep submicron technology scaling. The EOP metric also reduces from 2.81 pJ/bit to 0.093 pJ/bit, confirming energy-efficient operation at the 45 nm node.

Overall, the data clearly indicates that both 8-bit and 16-bit MAC architectures achieve substantial gains in speed, area efficiency, and energy performance with progressive technology scaling. The observed improvements validate the scalability and technology adaptability of the proposed MAC architecture across deep submicron nodes.

**Table 7:** Frontend design performance parameters of 8-bit and 16-bit MAC units.

Parameter	8-bit MAC			16-bit MAC		
	180 nm technology node	90 nm technology node	45 nm technology node	180 nm technology node	90 nm technology node	45 nm technology node
Propagation delay(ns)	9.758	8.412	1.12	17.738	11.094	2.282
Power consumption(mW)	1.412	0.339	0.057	2.54	0.861	0.659
Area consumption(µm^2^)	19865.261	5927.284	1702.22	72535.479	21349.878	8418.84
Power delay product (pJ)	13.778	2.85	0.069	45.05	9.55	1.503
Energy delay product (pJ. ns)	134	23.98	0.072	799.17	105.97	3.432
Energy per operation(pJ/bit)	1.72	0.356	0.008	2.81	0.6	0.093

The performance of the 16-bit Multiply–Accumulate (MAC) unit implemented using the Grouping and Decomposition (GD) multiplier with a carry-skip adder has been compared against five existing architectures synthesized at the 90 nm technology node. The compared designs include Karatsuba-based MACs (RCA and CS variants) and Wallace tree-based MACs employing Brent–Kung, Ripple Carry, and Carry Save adders. The results presented in Table 8 clearly demonstrate the superior overall efficiency of the proposed GD-based MAC architecture across key design metrics such as delay, power, and energy efficiency.

In terms of propagation delay, the GD-based MAC exhibits the minimum delay of 11.094 ns, achieving up to a 70% reduction compared to the slowest architecture, Karatsuba-RCA (37.09 ns). This significant improvement can be attributed to the inherent parallelism in the GD multiplier, which divides the input operands into smaller sub-blocks and processes them concurrently. Additionally, the carry-skip adder minimizes delay by bypassing unnecessary carry propagation stages, leading to faster accumulation and reduced critical path delay. In contrast, Karatsuba- and Wallace-based architectures exhibit higher delay due to multiple sequential partial-product recombinations and longer adder carry paths.

From a power consumption perspective, the GD-based MAC achieves a balanced trade-off between computational speed and energy use, consuming only 0.861 mW. While slightly higher than the lowest value reported for Wallace–Brent Kung (0.620 mW), it remains lower than most other architectures, including Karatsuba-CS (0.900 mW) and Wallace-CS (1.020 mW). This indicates that the decomposition-based multiplier is power-efficient despite its higher transistor count, owing to optimized switching activity and reduced signal transitions within the partial-product network.

**Table 8:** Frontend design performance parameters of 16-bit GD based MAC against other architectures in 90 nm technology node.

Parameter	Karatsuba Multiplier and Ripple carry adder (Karatsuba-RCA)	Karatsuba Multiplier and carry save adder (Karatsuba-CS)	Wallace tree multiplier and Brent Kung adder (Wallace-Brentkung)	Wallace tree multiplier and Ripple carry adder (Wallace-RCA)	Wallace tree multiplier and carry save adder (Wallace-CS)	GD multiplier with carry skip adder (GD based MAC)
Propagation delay(ns)	37.09	36.59	24.12	29.43	26.32	11.094
Power Consumption(mw)	0.470	0.900	0.620	0.800	1.020	0.861
Area consumption (µm^2^)	8100	8900	8600	9400	10500	21349.878
Power delay product (pJ)	17.43	32.93	14.95	23.54	26.85	9.55
Energy delay product (pJ. ns)	646.56	1204.94	360.700	692.89	706.59	105.97
Energy per operation (pJ/bit)	1.089	2.058	0.93	1.47	1.68	0.6

Regarding energy-performance metrics, the GD-based MAC achieves the lowest Power Delay Product (PDP) of 9.55 pJ, indicating superior power–speed efficiency compared to all other designs. Similarly, the Energy Delay Product (EDP) is drastically reduced to 105.97 pJ·ns, outperforming traditional approaches by over 90% relative to Karatsuba-CS (1204.94 pJ·ns) and by 85% compared to Wallace-CS (706.59 pJ·ns). The Energy per Operation (EOP) further validates the efficiency of the proposed architecture, reaching 0.6 pJ/bit, which represents an improvement of 35%–70% over the compared MAC designs.

Although the area utilization of the proposed GD-based MAC (21349.878 μm²) is higher than that of other architectures, this increase primarily arises from the additional decomposition and control logic required to introduce parallelism within the MAC structure. This architectural complexity enables substantial improvements in delay and energy efficiency, effectively balancing performance and area to achieve an optimal trade-off for high-throughput DSP applications.

However, it is acknowledged that the observed area overhead may limit the suitability of the proposed design for area-constrained or resource-limited platforms, such as edge devices and embedded signal processors, where silicon footprint is a critical constraint. In such environments, compactness often takes precedence over maximum speed, making the current implementation more appropriate for performance-oriented DSP and AI accelerator platforms. To address this limitation, future work will focus on incorporating area-optimization techniques such as Booth encoding, compressor-based partial-product reduction, and resource sharing among submodules to mitigate area growth without significantly compromising parallelism or computational efficiency. These refinements are expected to enhance scalability and extend the GD-based MAC architecture toward low-power, edge-compatible implementations while retaining its high-speed advantages.

Overall, the GD multiplier with a carry-skip adder demonstrates significant advantages in speed, energy efficiency, and scalability over conventional Karatsuba- and Wallace-tree-based MAC architectures. Although it incurs moderate area overhead, the overall improvement in delay and energy parameters establishes it as a highly optimized, low-power solution for advanced 16-bit MAC implementations, making it particularly suitable for energy-efficient and high-throughput DSP and AI processing systems.

**Fig. 13:**
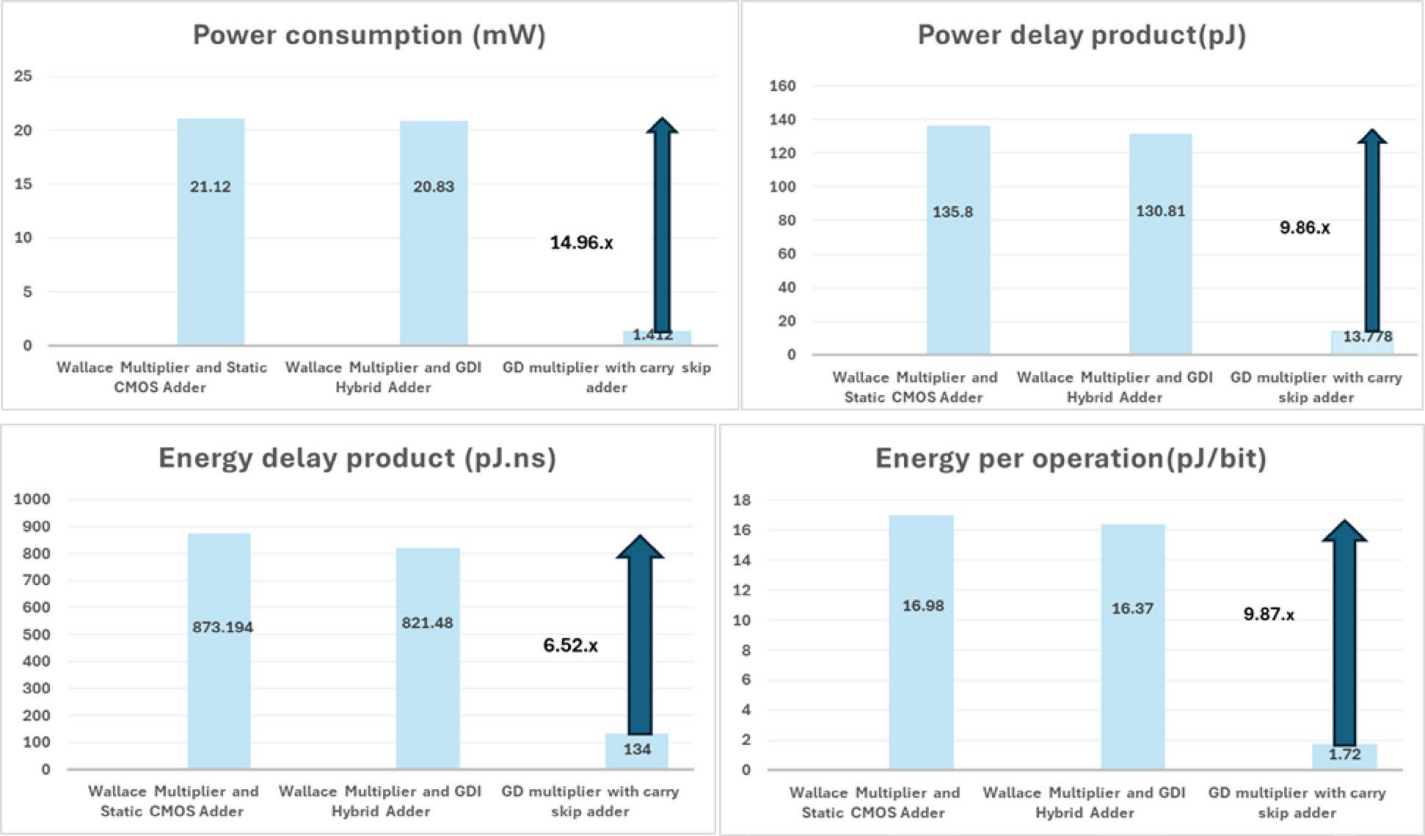
Power consumption, Power delay product, Energy delay product and Energy per operation of GD based 8 × 8 MAC architecture with other existing MAC architecture in 180 nm technology node.

Figure 13 illustrates the performance comparisons of 8 × 8 GD based MAC design in 180 nm technology against other existing designs^5^. The GD based 8 × 8 MAC architecture (Designed using GD multiplier with carry skip adder) consumes 14.96 times less power consumption, offers 9.86 times improved PDP and EOP, and 6.52 times improved EDP than the existing MAC architectures.

### Backend design of 16-bit MAC unit

The physical design of the 16-bit GD-based MAC unit, developed using the proposed automated HDL Verilog script, was implemented using the Cadence^®^ Innovus tool for both 180 nm and 90 nm technology nodes. Partitioning and floor planning were performed based on the required number of input cycles (M) and worst-case timing analysis. Input and output port placements were optimized to minimize crosstalk and simultaneous switching noise, ensuring robust signal integrity. Via-redundancy routing^39^ procedure was implemented using six metal layers, with efforts directed toward minimizing congestion scores and enhancing routing reliability.

The post-layout simulation performance metrics are detailed in Table 9. At the 180 nm technology node, the backend implementation of the 16-bit GD-based MAC unit achieves an output computation time of 18.02 ns for three input sequences, with an average power consumption of 5.2 mW. The corresponding Power-Delay Product (PDP) is calculated as 93.70 × 10⁻¹² J. In contrast, the same architecture implemented at the 90 nm technology node shows significant improvements: it requires only 12.55 ns for computation, with a reduced average power consumption of 1.004 mW and a much lower PDP of 12.60 × 10⁻¹² J. These results clearly indicate that the GD-based MAC architecture benefits substantially from technology scaling, delivering enhanced performance, reduced power consumption, and improved area efficiency.

**Table 9:** 16-bit GD based MAC architecture backend design performance parameters.

Design (16-bit MAC)	Maximum delay (ns)	Power consumption(mW)	Power delay product(pJ)	Area(µm^2^)
180 nm technology node	18.02	5.2	93.70	76311.75
90 nm technology node	12.55	1.004	12.60	22417.37

## Conclusion

This research introduces an automated and scalable HDL generation framework for the design of Multiply-Accumulate (MAC) units aimed at high-speed and energy-efficient applications. Utilizing a Python-based script, the framework generates synthesizable Verilog code for any user-defined bit-width, significantly reducing manual design effort and accelerating development time. The proposed MAC architecture integrates a GD multiplier with a carry skip adder to enable parallel processing and reduce carry propagation delays, thereby enhancing computational efficiency. The correctness and scalability of the proposed framework are verified through functional simulation, RTL synthesis, and backend physical design implementation using various GPDK technology nodes. Comprehensive frontend and backend evaluations confirm that the GD-based MAC design outperforms conventional architectures across various performance metrics, regardless of bit-width or technology node. The results substantiate the effectiveness of the proposed framework in enabling rapid prototyping of MAC units for DSP and AI applications, while also laying the foundation for future advancements in low-power, flexible hardware accelerators.

## Data Availability

All data generated or analyzed during this study are included in this article.
